# Multiple liver metastases of pancreatic solid pseudopapillary tumor treated with resection following chemotherapy and transcatheter arterial embolization: A case report

**DOI:** 10.3892/ol.2015.2967

**Published:** 2015-02-16

**Authors:** HIDEHIRO TAJIMA, HIROYUKI TAKAMURA, HIROHISA KITAGAWA, AKIRA NAKAYAMA, MASATOSHI SHOJI, TOSHIFUMI WATANABE, TOMOYA TSUKADA, SHINICHI NAKANUMA, KOICHI OKAMOTO, SEISHO SAKAI, JUN KINOSHITA, ISAMU MAKINO, KEISHI NAKAMURA, HIRONORI HAYASHI, KATSUNOBU OYAMA, MASAFUMI INOKUCHI, HISATOSHI NAKAGAWARA, TOMOHARU MIYASHITA, ITASU NINOMIYA, SACHIO FUSHIDA, TAKASHI FUJIMURA, TOMOHIKO WAKAYAMA, SHOICHI ISEKI, HIROKO IKEDA, TETSUO OHTA

**Affiliations:** 1 Department of Gastroenterologic Surgery, Kanazawa University, Graduate School of Medical Science, Kanazawa 920-8641, Japan; 2 Department of Histology and Embryology, Kanazawa University, Graduate School of Medical Science, Kanazawa 920-8641, Japan; 3 Division of Pathology, Kanazawa University, Graduate School of Medical Science, Kanazawa 920-8641, Japan

**Keywords:** solid pseudopapillary tumor, liver metastasis, resection, hepatic arterial infusion, transarterial tumor embolization, chemotherapy

## Abstract

A 33-year-old female was diagnosed with a solid pseudopapillary tumor (SPT) of the pancreas and multiple liver metastases at the Department of Gastroenterological Surgery, Ishikawa Prefectural Central Hospital (Kanazawa, Japan). Distal pancreatectomy and postoperative systemic chemotherapy with gemcitabine (GEM) and S-1, an oral fluoropyrimidine derivative, was administered, however, liver metastases became enlarged and local recurrence occurred. Therefore, the patient was referred to the Department of Gastroenterologic Surgery at the Graduate School of Medicine (Kanazawa, Japan) for hepatic arterial infusion (HAI) chemotherapy. Oral S-1 (80 mg/m^2^) was administered as well as HAI chemotherapy with GEM (1,000 mg/standard liver volume). Following 18 cycles, tumor sizes were reduced and 18-fluorodeoxyglucose positron emission tomography (^18^FDG-PET) examination revealed obvious reduction of tumor FDG uptake. Transarterial tumor embolization (TAE) was performed for the previously unresectable right subphrenic liver tumor, and the other tumors were surgically resected. The resected tumors were diagnosed as liver metastases and a local recurrence of SPT in the postoperative pathological examination, which revealed that the resected tumors were composed of sheets of bland cells, which were positive for CD10, CD56, vimentin, neuron-specific enolase and α-antitrypsin. The postoperative course was uneventful, and the patient is currently under observation at an outpatient clinic; postoperative adjuvant chemotherapy with oral S-1 has continued, and additional TAE is planned. In the future, if the middle segment of the liver becomes enlarged, surgery for the residual right lobe tumor may be possible. This case demonstrates one method of SPT treatment: Preoperative HAI chemotherapy with GEM, plus oral S-1 and TAE. If complete resection can be achieved, the majority of patients with SPT have a favorable prognosis. In patients with unresectable metastases from SPT, it is crucial to conduct systematic multimodal treatment to maximize treatment success.

## Introduction

Solid pseudopapillary tumor (SPT) is a rare, nonfunctional neoplasm of the pancreas that occurs most frequently in young females with a mean age of 25 years (90% of all cases occur in female patients) (1). The first case was reported by Frantz in 1959 (2). The tumor may occur anywhere in the pancreas, however, is most frequently identified in the pancreatic body and tail. Histopathologically, SPT is classically defined as a large and encapsulated mass composed of a mixture of cystic and solid areas. Intratumoral hemorrhage is frequent, and calcifications have been reported in ≤30% of cases (3–5). Although SPT is considered to be an indolent lesion with low malignant potential and a favorable prognosis following surgical resection, a number of cases of locally infiltrating and metastatic varieties, and post-surgical recurrences have been reported (1). In 1996, the World Health Organization renamed the tumor as SPT and reclassified it as a low-grade malignant tumor (6). The incidence of SPT is low, accounting for 1–2% of exocrine pancreatic tumors and 5% of cystic pancreatic neoplasms.

The current study reports a case of SPT with multiple liver metastases and local recurrence following distal pancreatectomy for the original lesion; the patient was treated with hepatic arterial infusion (HAI) chemotherapy, systemic chemotherapy, transarterial embolization (TAE), and surgical resection. Written informed consent was obtained from the patient.

## Case report

**Figure 1:**
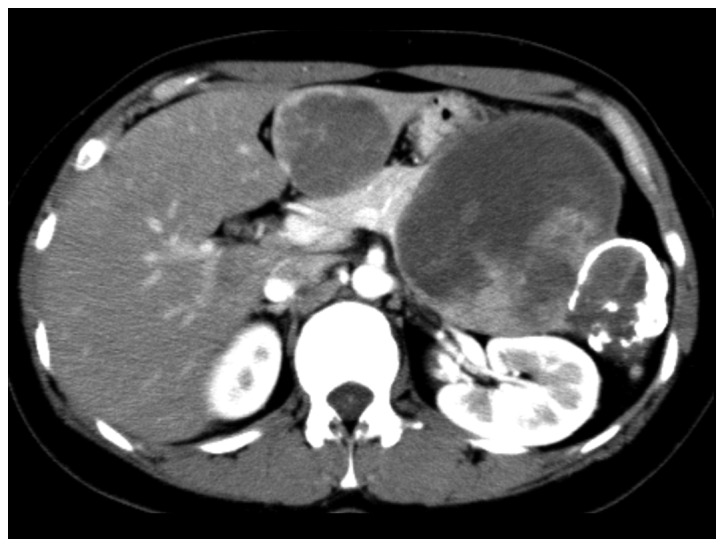
Abdominal computed tomography image following previous hospital admission. A cystic tumor of 10 cm in diameter in the pancreatic tail (with calcification) and multiple liver tumors were detected.

A 33-year-old female was admitted to the Department of Gastroenterological Surgery, Ishikawa Prefectural Central Hospital (Kanazawa, Japan) in December 2006 with abdominal pain and vomiting. A cystic lesion of 10 cm in diameter was detected in the tail of the pancreas as well as multiple liver tumors using abdominal computed tomography (CT) (Fig. 1). In January 2007, distal pancreatectomy was performed at Ishikawa Prefectural Central Hospital. The pancreatic tumor was diagnosed as SPT on postoperative pathological examination, which revealed that the resected tumors were composed of sheets of bland cells with oval to round nuclei and a focal pseudopapillary appearance. Furthermore, immunohistochemical analysis revealed positivity for CD10, CD56, vimentin, neuron-specific enolase (NSE) and α-antitrypsin. The multiple liver tumors were hypothesized to be SPT metastases. Following surgery, systemic chemotherapy with gemcitabine (GEM) and S-1, an oral fluoropyrimidine derivative, was administered. However, the liver metastases gradually enlarged, and the patient was referred to the Department of Gastroenterologic Surgery at the Graduate School of Medicine (Kanazawa, Japan) for hepatic arterial infusion (HAI) chemotherapy in September 2011.

**Figure 2:**
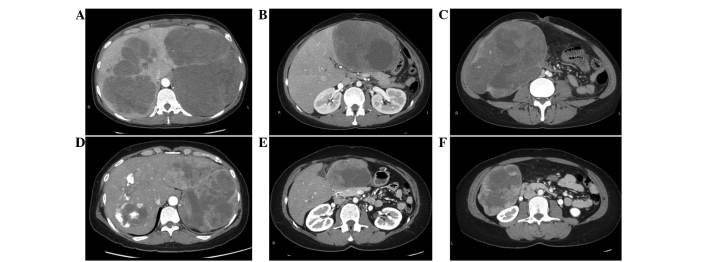
Abdominal CT images pre- and post-chemotherapy. (A) Abdominal CT showed recurrence of the local tumor following distal pancreatectomy in the form of a large tumor (15 cm in diameter) in the left subphrenic area, as well as liver metastases in the left lateral segment and anteroposterior segment. (B) The left lateral segment was occupied by a tumor, which was palpable in the upper abdomen. (C) A large mass of the lower posterior segment was palpable in the right lower quadrant of the abdomen. (D–F) Following combination therapy with gemcitabine hepatic arterial infusion, plus oral S-1 and transarterial embolization of the anteroposterior segment, the recurrent local tumor was slightly reduced in size and the liver metastases were obviously reduced. CT, computed tomography.

**Figure 3:**
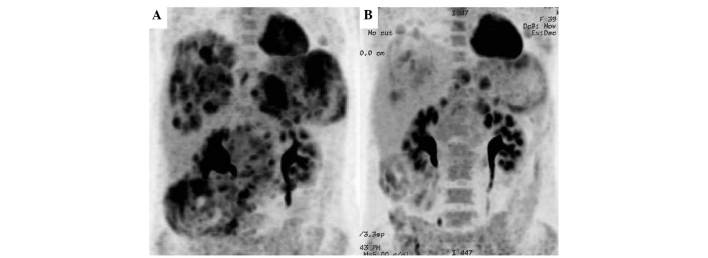
FDG-PET analysis. (A) Extremely high FDG uptake by the tumors was detected on FDG-PET analysis. (B) Following chemotherapy and transarterial embolization, a reduction of FDG uptake was observed. FDG-PET, 18-fluorodeoxyglucose-positron emission tomography.

Physical examination identified palpable hard masses of >10 cm in diameter around the umbilical area and right lower quadrant. Abdominal contrast-enhanced CT revealed multiple heterogeneous solid and cystic tumors in the liver, and a large tumor of 15 cm in diameter was identified in the left subphrenic area, indicating local recurrence following distal pancreatectomy (Fig. 2A). The lateral segment of the liver was infiltrated with tumors (Fig. 2B), and the 15-cm diameter tumor had been growing suspended from the posterior segment of the liver (Fig. 2C). Additionally, large tumors were identified in the right subphrenic area in the anteroposterior segment of the liver. Complete surgical resection of the tumors was considered to be impossible, as the tumors were located in close proximity to the major Glisson sheath (Fig. 2A). Examination with 18-fluorodeoxyglucose positron emission tomography (FDG-PET) revealed all of the tumors to have high FDG uptake, with maximum standardized uptake values (SUV max) of 7.7–8.8 (Fig. 3A).

As it was impossible to treat the subphrenic local recurrent tumor with HAI chemotherapy, systemic chemotherapy was used in combination. In October 2011, oral S-1 (80 mg/m^2^) and HAI with GEM (1,000 mg/standard liver volume) were initiated as described in a previous report (7). Following 18 cycles of HAI chemotherapy, the tumors exhibited a 26.3% reduction in size (Fig. 2D–F). Although this result was determined to be stable disease according to the Response Evaluation Criteria in Solid Tumors guidelines (8), FDG-PET examination revealed obvious reduction of tumoral FDG uptake, and the SUV max was 5.4 in the lesion with the highest uptake (Fig. 3B).

While chemotherapy was effective, a complete resection was not predicted to be successful in this situation, due to the involvement of the major Glisson sheath. For the right subphrenic tumor, transcatheter arterial embolization (TAE) was a viable treatment. However, TAE was likely to cause the other tumors to rupture due to post-treatment necrosis. Therefore, TAE was performed for the unresectable right subphrenic liver tumor occupying the anteroposterior segment, and the other tumors were surgically resected once the patient’s condition stabilized post-TAE.

**Figure 4:**
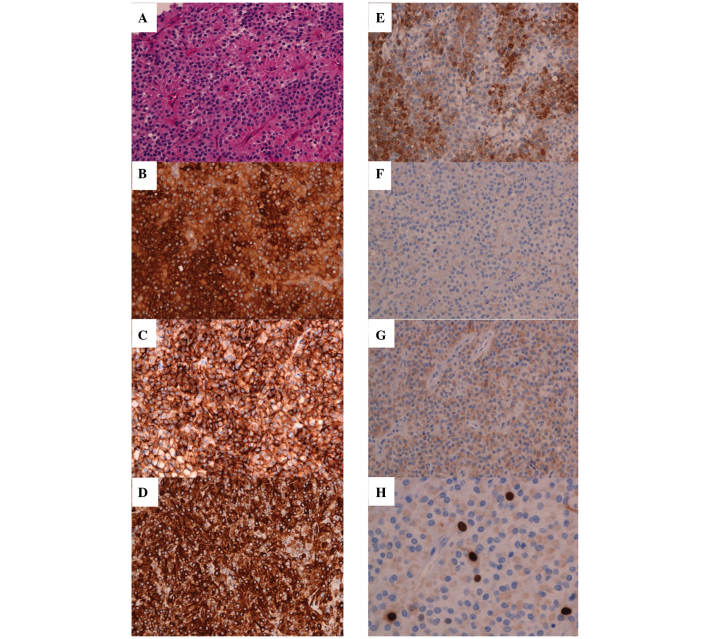
Histological features and immunohistochemical characteristics of the tumor. (A) Histological features include pseudopapillary architecture with fibrovascular stalks and small, uniform tumor cells with round nuclei. The tumor was characterized by positive staining for (B) α-antitrypsin, (C) CD56, (D) vimentin, and (E) NSE; and by weak staining for (F) chromogranin A and (G) synapthophysin. (H) The Ki-67 index was low (1–2%).

**Figure 5:**
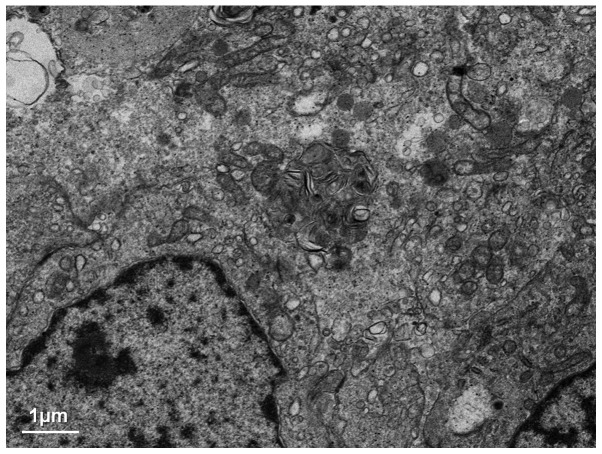
Electron microscopy: Dilated mitochondria and small vacuoles are seen. Large para-nuclear membrane-bound vacuoles and small vacuoles are observed.

The resected tumors were diagnosed as liver metastases and a local recurrence of SPT on postoperative pathological examination. The tumor specimen was a highly vascular lesion composed of sheets of bland cells with oval to round nuclei, moderate cytoplasm, ill-defined cell borders, and a focal pseudopapillary appearance (Fig. 4A). On immunohistochemical analysis, the tumor cells reacted positively for CD10, CD56, vimentin, NSE, and α-antitrypsin, however, the Ki-67 index was low (1–2%; Fig. 4B–H). Electron microscopy was performed to characterize the nature of the cytoplasmic vacuoles that appeared to be dilated or distended mitochondria; the remaining fractions appeared to be smooth endoplasmic reticulum, consistent with a previous report (Fig. 5). In a number of the small vacuoles, a few cristae could still be identified as mitochondrial, and a gradual transition from the normal mitochondria to those with attenuation of cristae was observed as well as loss of matrix.

The postoperative course was uneventful, and the patient is currently under monthly observation at an outpatient clinic and receiving adjuvant chemotherapy with oral S-1 (100 mg, every other day).

## Discussion

SPTs of the pancreas are uncommon low-malignant epithelial tumors that are typically identified in adolescent females. Metastatic disease is rare and only occurs in around 10–15% of patients (9–11). Previous studies have indicated the most frequently observed metastatic sites to be the liver and omentum (12). Resection of the primary pancreatic tumors or liver metastases has yielded excellent survival with an overall cure rate of >90% (13–16). The clinical features of SPT are non-specific and are often caused by compression from the tumor. Abdominal pain or discomfort is the most common symptom, followed by back pain, nausea, vomiting, weight loss, and diarrhea (1). A number of patients present with jaundice, upper gastrointestinal bleeding, or other rare symptoms; however, a considerable number of patients exhibit no symptoms, and SPT is identified incidentally during physical examination or on ultrasound, CT, or other imaging examinations (1). Although resection of the tumor yields a five-year survival rate of 97%, local recurrence or distant metastases occur in 10–15% of patients (1).

The primary morphological differential diagnosis for SPT is pancreatic neuroendocrine tumor. Traditionally, negative staining for neuroendocrine markers, in particular chromogranin A, has been considered crucial to this distinction. However, a recent study demonstrated aberrant nuclear staining for β-catenin and a loss of membranous expression of E-cadherin with aberrant nuclear localization of the cytoplasmic domain in all pancreatic SPT cases analyzed (17). In this study, the majority of pancreatic SPTs were also strongly positive for vimentin (100%), β-catenin nuclear stain (100%), CD10 (96%), progesterone receptor (79%), CD56 (75%), cytokeratin (28%), synapthophysin (26%), and chromogranin A (15%) (17). In the present case, CD10, CD56, and vimentin were strongly positive.

At present, no consistent clinical or histological criteria has been established to predict the biological behavior of SPT. Invasion of blood vessels, peritoneal infiltration, invasion of adjacent structures, a high degree of cellular polymorphism, and an elevated mitotic rate are characteristics proposed to be associated with metastases and recurrence. However, the absence of these features does not preclude malignant behavior. In the present case, the patient exhibited multiple liver metastases at initial presentation in the absence of malignant histological behavior.

The current study demonstrates that SPT is an indolent tumor with an excellent prognosis and that surgical resection is the mainstay of treatment, even in the presence of local invasion and extrapancreatic involvement. Indeed, involvement of the surgical margins (R1) does not appear to be associated with a poor outcome. In previously reported studies, ≤20% of the cases have exhibited liver metastases at the time of resection, however, the overall five-year survival rate remains >95% (11). In the present patient, as the liver metastases were diffuse, radical excision of these metastases was not possible. Therefore, combination therapy with TAE and chemotherapy was selected for treatment of the remnant liver tumor.

The value of chemotherapy for patients with SPT remains unknown, however, a number of anecdotal studies have reported its benefit (13,18–20) and lack of benefit (9,21,22). Two cases involving resectable tumors were subjected to chemotherapy with cisplatin and 5-fluorouracil (5-FU) (19) or GEM (20), but in the latter case, previous treatment with 5-FU and radiation had failed to decrease the tumor size. Radiotherapy is occasionally used for the treatment of unresectable tumors or as an adjuvant treatment following tumor resection (13). Two cases of radiosensitivity in unresectable tumors have been reported (23,24). Kanter *et al* reported the advantages of neoadjuvant chemotherapy with GEM for a large SPT (25). In another report, a patient who presented with a large SPT arising from the pancreatic body and tail (with gastric wall infiltration and para-aortic lymphadenopathy) was treated with GEM and cisplatin. During this therapy, the tumor regressed by >50%, with disappearance of the para-aortic lymphadenopathy and posterior gastric wall infiltration; the patient subsequently underwent full surgical resection (26).

In the current patient, oral S-1 and HAI with GEM were administered, which resulted in the reduction of tumor size on CT and an obvious reduction of FDG uptake on FDG-PET analysis. It was originally proposed that S-1 and GEM had been invalidated as treatment options based on their ineffective use at the previous hospital, however, it is possible that a sufficient amount had not been administered due to patient complaints and side effects. In the Department of Gastroenterologic Surgery at the Graduate School of Medicine, combination treatment with oral S-1 and HAI using GEM was effective. However, there is a possibility that oral S-1 administration alone was effective in treating the local recurrence at the left diaphragm, which was not reached by the arterial infusion of GEM but nonetheless decreased slightly in size. Therefore, oral S-1 was used for postoperative adjuvant chemotherapy.

Transarterial tumor embolization and transcatheter arterial chemoembolization are seldom used to treat SPT or similar pancreatic neoplasms (21,22,27). Among previous cases, one patient experienced a significant reduction in metastases of the right lobe (21), one succumbed to the disease following the procedure (27), and one patient’s disease remained unchanged (22). Radiofrequency ablation (RFA) is also a seldom-used modality (22,28,29). RFA is a safe and effective treatment for multiple unresectable liver metastases of SPT (29). However, incomplete RFA may induce dedifferentiation and epithelial-mesenchymal transition of the tumor (30,31). Therefore, it is advisable to limit the use of RFA to unresectable and small lesions.

In the current study, TAE was performed for residual liver tumors prior to surgical resection. Oral S-1 was subsequently administered as postoperative chemotherapy, and additional TAE is planned. In the future, if the central region of the liver enlarges, surgery for the residual right lobe tumor may become possible.

In conclusion, this case demonstrates one method of treatment for SPT: Preoperative chemotherapy with GEM HAI in combination with oral S-1 and TAE. If complete resection can be achieved, the majority of patients with SPT have a favorable prognosis. In patients with unresectable metastases from SPT, it is crucial to perform systematic multimodal treatment, with a combination of surgery, chemotherapy and interventional radiology, to maximize treatment success.
